# Text Messaging to Improve Attendance at Post-Operative Clinic Visits after Adult Male Circumcision for HIV Prevention: A Randomized Controlled Trial

**DOI:** 10.1371/journal.pone.0043832

**Published:** 2012-09-05

**Authors:** Thomas A. Odeny, Robert C. Bailey, Elizabeth A. Bukusi, Jane M. Simoni, Kenneth A. Tapia, Krista Yuhas, King K. Holmes, R. Scott McClelland

**Affiliations:** 1 Department of Epidemiology, University of Washington, Seattle, Washington, United States of America; 2 Center for Microbiology Research, Kenya Medical Research Institute, Nairobi, Kenya; 3 Chicago Developmental Center for AIDS Research, University of Illinois at Chicago, Chicago, Illinois, United States of America; 4 Department of Global Health, University of Washington, Seattle, Washington, United States of America; 5 Department of Obstetrics and Gynecology, University of Washington, Seattle, Washington, United States of America; 6 Department of Psychology, University of Washington, Seattle, Washington, United States of America; 7 Department of Medicine, University of Washington, Seattle, Washington, United States of America; 8 Center for AIDS and STD, University of Washington, Seattle, Washington, United States of America; Indiana University and Moi University, United States of America

## Abstract

**Background:**

Following male circumcision for HIV prevention, a high proportion of men fail to return for their scheduled seven-day post-operative visit. We evaluated the effect of short message service (SMS) text messages on attendance at this important visit.

**Methodology:**

We enrolled 1200 participants >18 years old in a two-arm, parallel, randomized controlled trial at 12 sites in Nyanza province, Kenya. Participants received daily SMS text messages for seven days (n = 600) or usual care (n = 600). The primary outcome was attendance at the scheduled seven-day post-operative visit. The primary analysis was by intention-to-treat.

**Principal Findings:**

Of participants receiving SMS, 387/592 (65.4%) returned, compared to 356/596 (59.7%) in the control group (relative risk [RR] = 1.09, 95% confidence interval [CI] 1.00–1.20; p = 0.04). Men who paid more than US$1.25 to travel to clinic were at higher risk for failure to return compared to those who spent ≤US$1.25 (adjusted relative risk [aRR] 1.35, 95% CI 1.15–1.58; p<0.001). Men with secondary or higher education had a lower risk of failure to return compared to those with primary or less education (aRR 0.87, 95% CI 0.74–1.01; p = 0.07).

**Conclusions:**

Text messaging resulted in a modest improvement in attendance at the 7-day post-operative clinic visit following adult male circumcision. Factors associated with failure to return were mainly structural, and included transportation costs and low educational level.

**Trial Registration:**

ClinicalTrials.gov NCT01186575

## Introduction

The epicentre of the HIV epidemic remains in sub-Saharan Africa, with an estimated 1.8 million new infections in 2009 [1]. This huge burden of disease continues to drive the agenda for research on efficacious interventions for HIV prevention. Male circumcision reduces the risk of HIV-1 acquisition by more than half [2]–[5]. The World Health Organisation (WHO) and the Joint United Nations Program on AIDS (UNAIDS) recommend that “*male circumcision should be considered an efficacious intervention for HIV prevention in countries and regions with heterosexual epidemics, high HIV and low male circumcision prevalence* [*6*] *.*” Programs are being scaled up to provide voluntary medical male circumcision in several countries in sub-Saharan Africa. Among these, Kenya has had the greatest success with the rollout of adult male circumcision, with more than 320,000 procedures performed by September, 2011 [7]–[9].

As male circumcision is widely and rapidly scaled-up for HIV prevention, follow-up of men after surgery must be assured [6]. WHO recommends that male circumcision services should include routine follow-up visits within seven days of the procedure [10]. This allows service providers to monitor wound healing, facilitating early detection and treatment of adverse events. Follow-up clinic visits are also an opportunity to reinforce post-operative care instructions including risk-reduction counselling and promotion of delayed resumption of sex. Unfortunately, most programs report that many men fail to return for scheduled post-operative visits. A male circumcision program in South Africa reported that only 67% of men returned for follow-up within one week after surgery [11]. In Kenya, the largest adult male circumcision program reports that only 43% of circumcised men return for follow-up within a week [12].

Interventions delivered via communications technology may be acceptable and efficacious for supporting medical service delivery [13]–[19]. For example, telephone reminders and text messages have led to increased clinic attendance for outpatients in developed and newly industrialized countries [15]–[19], and are acceptable for use as reminders for clinic return [20], [21]. The rapid increase in mobile telephone connections in developing countries presents an affordable and far-reaching avenue to improve health outcomes. In sub-Saharan Africa, text messages sent using the short messaging service (SMS) have been efficacious in improving adherence to antiretroviral treatment and achieving HIV viral load suppression [22], [23]. Text messages are also effective for improving uptake of public health interventions such as vaccination and smoking cessation [13], [14]. As programs for male circumcision for HIV prevention expand to provide services to hundreds of thousands of men in sub-Saharan Africa, it will be important to investigate low-cost, evidence-based strategies to improve health outcomes after male circumcision.

Our objective was to determine the effect of regular, context-sensitive text messages sent to men after undergoing circumcision, on their attendance at the scheduled seven-day post-operative clinic visit.

## Methods

The protocol for this trial and supporting CONSORT checklist are available as supporting information; see Checklist S1 and Protocol S1.

### Ethics statement

This study was approved by the Kenya Medical Research Institute's Ethical Review Committee, the University of Washington's Human Subjects Division, and Institutional Review Board #3 at the University of Illinois at Chicago. All study participants provided written informed consent prior to enrolment.

### Trial design and participants

We conducted a two-arm, parallel, randomized controlled trial in which the interventions were allocated in a 1∶1 ratio. Participants were recruited from among men undergoing circumcision at any of 12 sites in Nyanza province, Kenya. Nyanza province has the highest HIV (14.9%) and lowest male circumcision (45%) prevalences in the country [24], [25], and is the primary region where male circumcision for HIV prevention is being scaled up by the Kenya Ministry of Health. Study sites were clinics operating under the Ministry of Health, and supported by the Nyanza Reproductive Health Society (nine sites), Family AIDS Care and Education Services (one site), and Impact Research and Development Organization (two sites). Men eligible for enrolment were 18 years of age or older, had undergone circumcision on the day of screening, owned a mobile phone, had the phone in their possession at the time of enrolment, and were able and willing to respond to a questionnaire administered by phone 42 days after circumcision. Men who reported prior or on-going participation in any other research study were ineligible.

Between September 2010 and April 2011, 1,200 men were randomly assigned to receive either the intervention or standard care. Men who had undergone circumcision were approached by study staff during the 30-minute post-operative recovery period. The purpose of the study was described, and those who agreed to be screened were asked questions to determine eligibility. For eligible men, study procedures were described in detail. Those interested in participating were invited to provide written informed consent prior to enrolment.

### Randomization and masking

A biostatistician in Seattle, who was not involved in any other aspect of study implementation, developed the randomization sequence. A block randomization scheme with variable blocks of size 4–16 was generated using Stata ralloc.ado module v3.5.2 [26]. Randomization was stratified by clinic. Investigators and study staff were blinded to the block number, block size, and sequence in the block. Individual participant randomization envelopes were shipped from Seattle to Kisumu, while the key to intervention assignments was retained in Seattle.

Participants were assigned to intervention arms using pre-prepared sequentially numbered, sealed, opaque envelopes containing group assignment. Study staff issued the next envelope in the series. Because of the nature of the intervention, it was not possible to mask participants to group assignments. However, clinicians and nurses performing the circumcision procedure and follow-up were not aware of study group assignment.

### Procedures

Eligible men were interviewed for baseline characteristics, then randomized to receive either the intervention (text message) or control condition (no text message). All participants' mobile phone numbers were confirmed using a text message-based registration system. Participants were asked to send a text message from their phone to a phone number linked to the study's automated software. Each participant's phone was credited with airtime worth twenty Kenya shillings (about US$0.25) to compensate for the cost of sending this text message. No other reimbursement was given. Because our text messaging system was one-way, there was no additional cost borne by participants. For all participants, the initial text message sent to the automated study software contained the participant site and study number. For participants randomized to the intervention arm, this message also included the preferred time of day and language (English, Kiswahili or Dholuo).

We used an automated text messaging software developed in conjunction with Dimagi Inc. (Charlestown, MA), utilizing their RapidSMS platform, an open source framework for SMS and web integrated applications [27]. Functionality was built into RapidSMS to allow it to send pre-programmed text messages to the intervention group at a selected time of day, and in the desired language. For the first seven days after circumcision, daily text messages were sent to all participants in the intervention arm. The messages were adapted from a pilot study conducted in Nyanza province to assess their acceptability among circumcised men (Table 1).

**Table 1:** Intervention text messages.

Post-op Day	Messages (English)
1	If u r not the intended recipient of this Male Circumcision (MC) message, please text STOP to 0722819835 and you will not receive future messages. Thank you.
1	This is your MC provider. It is normal to feel a bit of pain and swelling, but if there is severe swelling, bleeding or pain please come back to the clinic.
2	This is your MC provider. Remember do not allow water to soak the dressing before removal on the 3^rd^ day.
3	Remove the dressing today. Make sure you review the post-op instructions and use the blade provided. Throw away the blade after use.
4	This is your MC provider. Always keep the genital area dry n clean to avoid infection. Do not apply any ointment or creams that are not prescribed by the clinic
5	This is your MC provider. If you feel heavy pain, swelling, bleeding, or any signs of infection please consult the clinic.
6	This is your MC provider. Don't forget to come back to the clinic for your day 7 follow-up visit. You will be checked to be certain the healing is going well.
7	This is your MC provider. See you at the clinic today for your follow-up visit.

Following the Kenya national guidelines for male circumcision services, men were asked to visit the clinic at seven days post-procedure. All procedures were documented in the participant's clinic records. The primary outcome was attendance at the seven-day post-operative clinic visit, determined by abstraction of clinic data from this record. Men were considered to have attended the seven-day post-operative visit on time if they returned within three days before or after the scheduled seven-day visit. Data abstracted from clinic records included the visit date, type of visit (routine/client-initiated/physician-scheduled), and the presence and severity (mild/moderate/severe) of adverse events. Twelve (1%) participants whose clinic records could not be located after an extensive search were considered lost to follow-up.

### Sample size

We assumed that 43% of men in the control group would return for the day seven visit, as observed in our Kenya male circumcision program. To provide at least 90% power to detect a relative risk (RR) between the two arms of 1.22 or larger for return to clinic (equivalent to an increase from 43% to 52.5%), we needed to enrol 1200 men (600 in each arm).

### Statistical analysis

All tests were two-sided, with a significance level of 0.05. All analyses were performed using Stata IC v10, according to a written statistical analysis plan that was established before evaluating the un-blinded data.

The primary analysis followed the intention-to-treat principle and was unadjusted. The SMS intervention versus control arms were compared using a chi-square test. Relative risks and confidence intervals (CIs) were calculated using Poisson regression with robust error variance [28]. For the absolute effect size, we computed the risk difference and the number needed to treat.

Among men who returned for the day seven visit, we also compared the proportion with circumcision-related adverse events, and present this RR with its 95% CI.

We performed secondary analyses estimating associations between other covariates and failure to return for the day seven visit using adjusted Poisson regression. Baseline characteristics significant at p<0.10 in unadjusted models were examined in a preliminary adjusted model; characteristics significant at p<0.10 were retained in this final adjusted model.

### Sensitivity and subgroup analyses

Baseline characteristics were assessed for imbalance between study arms. We also compared baseline characteristics among men retained in the study versus those whose clinic records could not be found. Sensitivity analyses were performed in which we considered the 12 men with missing clinic records as failures to return.

## Results

**Figure 1:**
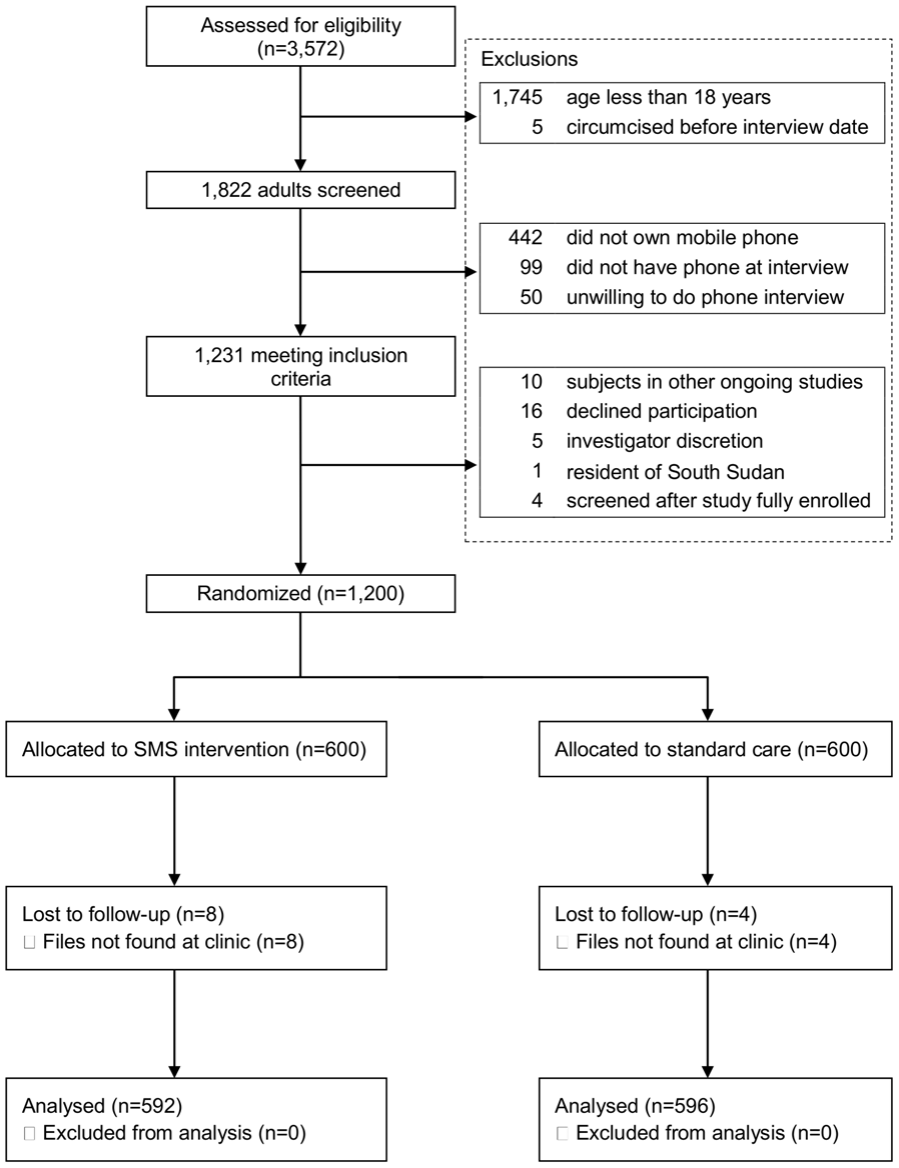
Trial profile.

Between September 2010 and April 2011, we screened 3,572 men who had undergone circumcision at 12 sites. The trial profile is shown in Figure 1. Among those screened, 1,745 (49%) were less than 18 years old and 5 (0.1%) were circumcised before the interview date. Of the remaining 1,822 adults, 442 (24%) did not own a phone, 99 (5%) did not have their phone with them at screening, and 50 (3%) were unwilling to respond to a phone interview. Eligible participants were randomized to either the intervention group (N = 600), or the control group (N = 600). Follow-up was completed in June 2011.

Baseline characteristics of the participants are presented in Table 2. The median age was 24.9 years (inter-quartile range [IQR] 21.5–30.7). The majority of participants were from the Luo ethnic group (1,153; 96.3%). Most of the participants (N = 870, 72.5%) reported at least one sexual partner in the preceding month. A sizable majority (N = 1093, 91%) had been tested for HIV infection. The median age at first sex was 16.0 years (IQR 14.0–18.0), and 672 (56%) participants were married or had a regular live-in partner. The median time taken to travel to and from the clinic was one hour (IQR 0.5–1.0), and the median time taken away from work in order to attend clinic was 3.0 hours (IQR 0.0–6.0). The median cost of travelling to and from the clinic was US$0.75 (IQR 0.38–1.25). There were minor differences in the proportions of men reporting zero versus one partner in the past month in the intervention versus control arms. However, the proportion reporting multiple partners was similar. Other baseline characteristics were similar in both study arms.

**Table 2:** Baseline Characteristics.

	SMS group	Control group
	(N = 600)	(N = 600)
Characteristic	n (%)	n (%)
Age (years) - median (IQR)	25.0 (21.4–30.7)	24.8 (21.5–30.5)
Luo ethnic group (versus other)	575 (96.2%)	578 (96.5%)
Employed	251 (42.0%)	264 (44.0%)
Study site		
UNIM Project	272 (45.3%)	270 (45.0%)
New Nyanza Provincial General Hospital	119 (19.8%)	118 (19.7%)
Tuungane Youth Project, Kisumu	79 (13.2%)	78 (13.0%)
Kombewa District Hospital	56 (9.3%)	56 (9.3%)
Tuungane Youth Project, Nyando	34 (5.7%)	33 (5.5%)
Chulaimbo District Hospital	18 (3.0%)	20 (3.3%)
Kisumu District Hospital	11 (1.8%)	11 (1.8%)
Siriba Health Center	7 (1.2%)	9 (1.5%)
Manyuanda Health Center	2 (0.3%)	2 (0.3%)
Ratta Health Center	1 (0.2%)	2 (0.3%)
Bodi Health Center	0 (0.0%)	1 (0.2%)
Barkorwa Health Center	1 (0.2%)	0 (0.0%)
Number of sex partners in previous month		
0	147 (24.5%)	183 (30.5%)
1	370 (61.7%)	335 (55.8%)
2+	83 (13.8%)	82 (13.7%)
Ever tested for HIV (versus never tested)	542 (90.5%)	551 (91.8%)
Condoms offered on day of circumcision	12 (2.0%)	10 (1.7%)
Age at first sex - median (IQR)	17.0 (14.0–18.0)	16.0 (14.0–18.0)
Travel time to/from clinic (hours) – median (IQR)	1.0 (0.5–1.5)	1.0 (0.5–1.0)
Transport cost to/from clinic (US$) – median (IQR)	0.75 (0.5–1.25)	0.75 (0.25–1.25)
Time away from work (hours) – median (IQR)	3.0 (0.0–6.0)	2.3 (0.0–6.0)
Education		
No level	20 (3.3%)	10 (1.7%)
Primary	154 (25.7%)	161 (26.9%)
Secondary	264 (44.1%)	245 (40.9%)
Post-secondary	161 (26.9%)	183 (30.6%)
Marital Status		
Not married, without regular live-in partner	252 (42.1%)	267 (44.5%)
Not married, with a regular live-in partner	111 (18.5%)	93 (15.5%)
Married, not living with wife	35 (5.8%)	44 (7.3%)
Married, living with wife	198 (33.1%)	191 (31.8%)
Other	3 (0.5%)	5 (0.8%)
Referral source		
Self	402 (67.1%)	396 (66.0%)
Parent	5 (0.8%)	7 (1.2%)
Friend	85 (14.2%)	100 (16.7%)
Voluntary Counselling and Testing	3 (0.5%)	3 (0.5%)
Community Health Worker	10 (1.7%)	7 (1.2%)
Other	94 (15.7%)	87 (14.5%)

IQR, interquartile range; UNIM, Universities of Nairobi, Illinois, and Manitoba.

Outcome data were available for 592 (98.7%) participants in the intervention group, and 596 (99.3%) in the control group. Overall, 743 men (62.5%) returned at day 7. In the intent-to-treat analysis, the proportion of men who returned was higher in the intervention group (387/592; 65.4%) than in the control group (356/596, 59.7%; RR 1.09, 95% CI 1.00–1.20; p = 0.04). In our sensitivity analysis where 12 participants whose clinic files could not be located to determine endpoints were treated as failure to return, the findings were similar, but were no longer statistically significant at the α = 0.05 level (RR 1.09, 95% CI 0.99–1.19; p = 0.07).

Among participants who returned for the day seven visit, 23/387 (6%) in the intervention group had an adverse event compared with 19/356 (5%) in the control group (RR 1.12, 95% CI 0.6–2.1; p = 0.7).

The association between different factors and men's risk of failure to return for follow-up are presented in Table 3. Men who paid more than 100 Kenyan shillings (US$1.25) to travel to and from the clinic had a higher risk of failure to return compared to those who spent ≤100 shillings (adjusted relative risk [aRR] 1.35, 95% CI 1.15–1.58; p<0.001). Men with secondary or higher education had a lower risk of missing their follow-up visit compared to those with primary or less education (aRR 0.87, 95% CI 0.74–1.01; p = 0.07), although this association was not statistically significant at the α = 0.05 level.

**Table 3:** Relative risk regression analysis of predictors of failure to return for the day 7 post-operative clinic visit.

	Failed to return	Unadjusted		Adjusted	
	N (%)	RR [95% CI]	p-value	RR [95% CI]	p-value
Study arm					
Control	240/596 (40.3%)	1 (ref)	—	1 (ref)	—
SMS	205/592 (34.6%)	0.86 [0.74–1.00]	0.04	0.86 [0.74–1.00]	0.04
Transport cost to/from clinic (US$)					
< = US$1.25	324/929 (34.9%)	1 (ref)	—	1 (ref)	—
>US$1.25	121/258 (46.9%)	1.34 [1.15–1.57]	<0.001	1.35 [1.15–1.58]	<0.001
Education level					
Primary or less	142/342 (41.5%)	1 (ref)	—	1 (ref)	—
Secondary or higher	302/844 (35.8%)	0.86 [0.74–1.01]	0.06	0.87 [0.74–1.01]	0.07

RR, relative risk; SMS, short message service.

## Discussion

In this randomized trial, SMS text messaging led to a modest increase in attendance at the seven-day post-operative clinic visit compared to a control condition with standard care. In the intent-to-treat analysis, 65% of men returned in the intervention arm compared to 60% in the control arm.

Our findings are consistent with those of studies evaluating text messaging and phone reminders for adherence to clinic visits for other medical conditions [15]–[19], although our effect size was smaller in magnitude. To our knowledge, this was the first randomized trial to evaluate SMS to improve clinic attendance in a resource-limited setting.

We found that return for the scheduled seven-day post-operative clinic visit was associated with lower travel cost related to clinic attendance. Men who incur a higher cost may not be willing to spend money on a return visit. These findings suggest that male circumcision programs might improve follow-up by providing other interventions for such groups of men, such as reimbursement of transport costs or mobile clinics to make services more readily accessible. We also found a statistical trend (p<0.10) suggesting that having less than high school education was independently associated with failure to return for the seven-day clinic visit. Additional or modified communications for men with low levels of education might help to address this problem.Alternatively, it is possible that the association between low levels of education and failure to return for follow-up may have more to do with lack of money for transportation in the less educated group compared to those with higher educational attainment.

Despite the randomized trial design, the findings from this study must be interpreted in the context of a number of limitations. First, 49% of those screened were excluded because they were less than 18 years old. As a result, it is difficult to generalize these results to this large population of younger men presenting for male circumcision. Second, we excluded men who did not own or have phones with them at the time of circumcision. No data were collected from these men, as they were not enrolled. Thus, it was not possible to directly compare men who had mobile phones to men who did not. This could limit generalizability and the impact of text messaging in the context of widespread scale-up of male circumcision interventions for HIV prevention. However, mobile phone access is rapidly expanding. In Kenya, with a population of 38 million people, there were 22 million mobile phone subscribers by September 2010 [29], with 63% of all Kenyan households owning at least one phone [30]. Third, it is common for people to change mobile phone numbers, and those who did so were unable to inform the study team of this change. Fourth, our SMS software was not programmed to request delivery notifications, and so we were unable to verify whether messages were received. It is possible that a number of men in the intervention arm did not receive the intervention if their phones could not be reached. Finally, the absolute increase of 5.7% in the proportion returning to clinic was a smaller change than our study was initially powered to detect. Nonetheless, we were able to detect a modest but statistically significant increase in follow-up with SMS text messages after male circumcision, due to our large sample size and high participant retention. Despite these limitations, we were able to demonstrate a modest increase in follow-up with SMS text messages after male circumcision.

Our finding that even in the SMS arm more than a third of participants failed to return parallels results from previous studies of medical interventions other than male circumcision, which have also reported that a high proportion of participants fail to return even when they receive messages [15]–[19]. Notably, rates of missed visits among both intervention and control participants in our study were lower than those typically reported by male circumcision programs in Kenya. It is possible that men behaved differently in a trial setting than in a non-research setting. Being in the study may have promoted return to clinic independent of the intervention (Hawthorne Effect) [31]. If this were the case, we may have under-estimated the reduction in missed visits due to the SMS intervention, because of a potent effect of the control condition.

Our SMS system was inexpensive. Except for the initial registration message, participants did not incur any costs. Our text messaging software was able to send out thousands of text messages per month at an average total cost of US$25 per month. The initial cost of setting up the system (US$5,000) would not be incurred again in the event of scale-up. Because the system is automated and software-based, neither additional human resources nor new physical infrastructure are needed. The SMS software easily works with different languages, and also works across geographical boundaries, limited only by the extent of mobile phone network coverage. Participant registration into the system took no more than two minutes per participant. Our low-cost intervention could easily be integrated into programs for male circumcision in Kenya, and likely in other sub-Saharan African countries. Similar studies using SMS or phone reminders demonstrated financial benefits of using SMS, including cost-effectiveness [15]–[19].

Our study utilized daily text messages, and it is possible that less frequent messages would be equally effective. Studies evaluating the use of SMS for antiretroviral adherence show that weekly messages may have higher efficacy than more frequent messages [22], [23]. While we believe that messages during the first few days after circumcision could be useful to promote a quick return to clinic at any sign of an adverse event, our study did not directly address this question. There is also a lack of published data on the relationship between missing the post-operative visit and the incidence of complications. Qualitative studies involving circumcised men could provide insight into the optimal timing and frequency of messages.

With on-going efforts to provide adult male circumcision to millions of men across sub-Saharan Africa, post-operative follow-up will be a key element of such large programs [10]. Of note, the number needed to treat was 18. That is, we would need to send text messages to 18 men for one additional man to return to clinic. This could lead to substantial benefits if applied to the millions of men willing to undergo circumcision for HIV prevention. At the same time, these findings show that a substantial proportion of men fail to return for post-operative visits even with daily messages intended to enhance follow-up. Understanding the additional reasons for failure to return will be essential in order to optimize follow-up rates. Future intervention strategies could benefit from combining SMS with behavioural and structural interventions to address transportation costs and message content for less educated men to further optimize return rates.

## Supporting Information

Protocol S1 **Trial protocol.**
(DOC)

Checklist S1 **CONSORT checklist.**
(DOC)

## References

[1] Global report: UNAIDS report on the global AIDS epidemic 2010. Available: http://www.unaids.org/globalreport/documents/20101123_GlobalReport_full_en.pdf Accessed Jul 08, 2011.

[2] Auvert B, Taljaard D, Lagarde E, Sobngwi-Tambekou J, Sitta R, et al (2005) Randomized, controlled intervention trial of male circumcision for reduction of HIV infection risk: the ANRS 1265 Trial. PLoS Med 2: e298. 16231970 10.1371/journal.pmed.0020298 PMC1262556

[3] Bailey RC, Moses S, Parker CB, Agot K, Maclean I, et al (2007) Male circumcision for HIV prevention in young men in Kisumu, Kenya: a randomised controlled trial. Lancet 369: 643–656. 17321310 10.1016/S0140-6736(07)60312-2

[4] Gray RH, Kigozi G, Serwadda D, Makumbi F, Watya S, et al (2007) Male circumcision for HIV prevention in men in Rakai, Uganda: a randomised trial. Lancet 369: 657–666. 17321311 10.1016/S0140-6736(07)60313-4

[5] Siegfried N, Muller M, Deeks JJ, Volmink J (2009) Male circumcision for prevention of heterosexual acquisition of HIV in men. Cochrane Database Syst Rev CD003362. 19370585 10.1002/14651858.CD003362.pub2 PMC11666075

[6] WHO/UNAIDS (2007) WHO/UNAIDS Technical Consultation Male Circumcision and HIV Prevention: Research Implications for Policy and Programming. Montreux, Switzerland.

[7] Herman-Roloff A, Llewellyn E, Obiero W, Agot K, Ndinya-Achola J, et al (2011) Implementing Voluntary Medical Male Circumcision for HIV Prevention in Nyanza Province, Kenya: Lessons Learned during the First Year. PLoS One 6: e18299. 21483697 10.1371/journal.pone.0018299 PMC3070734

[8] Clearinghouse on Male Circumcision for HIV Prevention. Available: http://www.malecircumcision.org/publications/male_circumcision_news.html Accessed Jul 08, 2011.

[9] Ochieng A (2011) Program Manager, Voluntary Medical Male Circumcision. Kenya National AIDS/STI Control Program: Personal Communication.

[10] WHO/UNAIDS/JHPIEGO. Manual for Male Circumcision under Local Anaesthesia. 2009; Available: http://www.malecircumcision.org/about/documents/MC_manual_local_anaesthesia.pdf Accessed Jul 8, 2011.

[11] Lissouba P, Taljaard D, Rech D, Doyle S, Shabangu D, et al (2010) A model for the roll-out of comprehensive adult male circumcision services in African low-income settings of high HIV incidence: the ANRS 12126 Bophelo Pele Project. PLoS Med 7: e1000309. 20652013 10.1371/journal.pmed.1000309 PMC2907271

[12] Bailey RC (2009) Personal Communication.

[13] Kharbanda EO, Stockwell MS, Fox HW, Andres R, Lara M, et al (2011) Text message reminders to promote human papillomavirus vaccination. Vaccine 29: 2537–2541. 21300094 10.1016/j.vaccine.2011.01.065

[14] Free C, Knight R, Robertson S, Whittaker R, Edwards P, et al (2011) Smoking cessation support delivered via mobile phone text messaging (txt2stop): a single-blind, randomised trial. Lancet 378: 49–55. 21722952 10.1016/S0140-6736(11)60701-0 PMC3143315

[15] Perron NJ, Dao MD, Kossovsky MP, Miserez V, Chuard C, et al (2010) Reduction of missed appointments at an urban primary care clinic: a randomised controlled study. BMC Fam Pract 11: 79. 20973950 10.1186/1471-2296-11-79 PMC2984453

[16] O'Brien G, Lazebnik R (1998) Telephone call reminders and attendance in an adolescent clinic. Pediatrics 101: E6. 10.1542/peds.101.6.e6 9606248

[17] Leong KC, Chen WS, Leong KW, Mastura I, Mimi O, et al (2006) The use of text messaging to improve attendance in primary care: a randomized controlled trial. Fam Pract 23: 699–705. 16916871 10.1093/fampra/cml044

[18] Liew SM, Tong SF, Lee VK, Ng CJ, Leong KC, et al (2009) Text messaging reminders to reduce non-attendance in chronic disease follow-up: a clinical trial. Br J Gen Pract 59: 916–920. 19712544 10.3399/bjgp09X472250 PMC2784529

[19] Downer SR, Meara JG, Da Costa AC, Sethuraman K (2006) SMS text messaging improves outpatient attendance. Aust Health Rev 30: 389–396. 16879098 10.1071/ah060389

[20] Person AK, Blain ML, Jiang H, Rasmussen PW, Stout JE (2011) Text messaging for enhancement of testing and treatment for tuberculosis, human immunodeficiency virus, and syphilis: a survey of attitudes toward cellular phones and healthcare. Telemed J E Health 17: 189–195. 21457085 10.1089/tmj.2010.0164 PMC3079163

[21] Foley J, O'Neill M (2009) Use of mobile telephone short message service (SMS) as a reminder: the effect on patient attendance. Eur Arch Paediatr Dent 10: 15–18. 19254521 10.1007/BF03262661

[22] Pop-Eleches C, Thirumurthy H, Habyarimana JP, Zivin JG, Goldstein MP, et al (2011) Mobile phone technologies improve adherence to antiretroviral treatment in a resource-limited setting: a randomized controlled trial of text message reminders. AIDS 25: 825–834. 21252632 10.1097/QAD.0b013e32834380c1 PMC3718389

[23] Lester RT, Ritvo P, Mills EJ, Kariri A, Karanja S, et al (2010) Effects of a mobile phone short message service on antiretroviral treatment adherence in Kenya (WelTel Kenya1): a randomised trial. Lancet 376: 1838–1845. 21071074 10.1016/S0140-6736(10)61997-6

[24] National AIDS and STI Control Programme MoH, Kenya *Kenya AIDS Indicator Survey 2007: Final Report* Nairobi, Kenya.

[25] (2010) Kenya Demographic and Health Survey 2008-09. Calverton, Maryland: Kenya National Bureau of Statistics (KNBS) and ICF Macro.

[26] Philip R (1997) RALLOC: Stata module to design randomized controlled trials. S319901 ed: Boston College Department of Economics.

[27] RapidSMS – An open source framework for SMS and Web integrated applications. Available: http://www.dimagi.com/rapidsms/ Accessed Jan 2010.

[28] Zou G (2004) A modified poisson regression approach to prospective studies with binary data. Am J Epidemiol 159: 702–706. 15033648 10.1093/aje/kwh090

[29] Communications Commission of Kenya Quarterly Sector Statistics Report (July–September 2010/2011). Available: http://www.cck.go.ke/news/2011/Mobile_subscribers.html Accessed Jul 08, 2011.

[30] (2010) The 2009 Kenya Population and Housing Census. Kenya National Bureau of Statistics.

[31] Franke RH, Kaul JD (1978) The Hawthorne Experiments: First Statistical Interpretation. American Sociological Review 43: 623–643.

