# Supplementary material for: Text Messaging to Improve Attendance at Post-Operative Clinic Visits after Adult Male Circumcision for HIV Prevention: A Randomized Controlled Trial
Source: PLoS One. 2012 Sep 5;7(9):e43832. doi: 10.1371/journal.pone.0043832 (PMC3434192; doi:10.1371/journal.pone.0043832)
Supplement: Protocol S1 — Trial protocol. (DOC) [file pone.0043832.s001.doc]

**ClinicalTrials.gov # NCT01186575**

**TEXT MESSAGING TO IMPROVE ADHERENCE TO POST-OPERATIVE CLINIC APPOINTMENTS AND REDUCE EARLY RESUMPTION OF SEXUAL INTERCOURSE AFTER ADULT MALE CIRCUMCISION: A RANDOMIZED CONTROLLED TRIAL**

**KEMRI/UW/UIC**

**INVESTIGATORS AND INSTITUTIONAL AFFILIATIONS**

| Principal Investigator: | Thomas A. Odeny, MBChB MPH Candidate Departments of Epidemiology and Global Health University of Washington  Medical Officer, Family AIDS Care and Educational Services, Research Care and Training Program, CMR, KEMRI |
| --- | --- |
| Co-Investigators: | Dr Elizabeth Anne Bukusi, MBChB, M.Med (ObGyn), MPH, PhD. Co-Director Research Care Training Program,(RCTP) Chief Research Officer, Center for Microbiology Research, KEMRI,  Honorary Lecturer, Department of Obstetrics and Gynecology, University of Nairobi,  Research Associate Professor Departments of Global Health and Obstetrics and Gynecology University of Washington |
|  | R. Scott McClelland, MD, MPH  Associate Professor of Medicine, Epidemiology, and Global Health  University of Washington |
|  | Jane M. Simoni, Ph.D. Professor, Department of Psychology University of Washington |
|  | King K. Holmes, MD, PhD William H. Foege Chair and Professor Department of Global Health Schools of Medicine and Public Health  Director, Center for AIDS and STD University of Washington |
|  | Robert C. Bailey, PhD, MPH Professor of Epidemiology School of Public Health University of Illinois at Chicago |

TABLE OF CONTENTS

[ABSTRACT 5](#__RefHeading___Toc258362263)

[LIST OF ACRONYMS 6](#__RefHeading___Toc258362264)

[1.0 INTRODUCTION 7](#__RefHeading___Toc258362265)

[1.1 Background 7](#__RefHeading___Toc258362266)

[1.2 Benefits of delayed resumption of sex after MC 7](#__RefHeading___Toc258362267)

[1.3 Counseling for MC 8](#__RefHeading___Toc258362268)

[2.0 RATIONALE 8](#__RefHeading___Toc258362269)

[2.1 Studies of medical and behavioral interventions delivered via phone 8](#__RefHeading___Toc258362270)

[2.2 Telephone reminders and adherence to clinic appointments 8](#__RefHeading___Toc258362271)

[2.3 Mobile telephone technology in developing countries 8](#__RefHeading___Toc258362272)

[2.4 Study Generalizability 9](#__RefHeading___Toc258362273)

[3.0 STUDY OBJECTIVES AND HYPOTHESES 10](#__RefHeading___Toc258362274)

[3.1 Specific Objectives 10](#__RefHeading___Toc258362275)

[3.2 Secondary Objective 10](#__RefHeading___Toc258362276)

[3.3 Study Outcome Measures 10](#__RefHeading___Toc258362277)

[4.0 STUDY DESIGN 11](#__RefHeading___Toc258362278)

[4.1 Overview of Study Design 11](#__RefHeading___Toc258362279)

[4.2 Study Setting 11](#__RefHeading___Toc258362280)

[4.3 Study Population 11](#__RefHeading___Toc258362281)

[4.4 Inclusion Criteria 11](#__RefHeading___Toc258362282)

[4.5 Exclusion criteria 12](#__RefHeading___Toc258362283)

[5.0 METHODOLOGY 12](#__RefHeading___Toc258362284)

[5.1 Treatment Assignment Procedures 12](#__RefHeading___Toc258362285)

[5.2 STUDY INTERVENTION/TEXT MESSAGING PROTOCOL 14](#__RefHeading___Toc258362286)

[6.0 STUDY CLINIC VISITS AND PROCEDURES 18](#__RefHeading___Toc258362287)

[6.1 Screening Visit 18](#__RefHeading___Toc258362288)

[6.2 Enrollment Visit 18](#__RefHeading___Toc258362289)

[6.3 Follow-up Visit 19](#__RefHeading___Toc258362290)

[6.4 Final Study Visit (Virtual Visit) 19](#__RefHeading___Toc258362291)

[6.5 Study Withdrawal for Participants 20](#__RefHeading___Toc258362292)

[7.0 STUDY ADVERSE EVENTS 20](#__RefHeading___Toc258362293)

[8.0 ETHICAL CONSIDERATIONS 21](#__RefHeading___Toc258362294)

[8.1 Informed Consent 21](#__RefHeading___Toc258362295)

[8.2 Ethical Approval 21](#__RefHeading___Toc258362296)

[8.3 Protecting Privacy and Confidentiality 21](#__RefHeading___Toc258362297)

[8.4 Potential Risks of Proposed Research to Study Subjects 21](#__RefHeading___Toc258362298)

[8.5 Potential Benefits of Proposed Research to Study Subjects and Others 22](#__RefHeading___Toc258362299)

[9.0 DATA COLLECTION AND MANAGEMENT 23](#__RefHeading___Toc258362300)

[9.1 Data Collection 23](#__RefHeading___Toc258362301)

[9.2 Data Storage and Security 23](#__RefHeading___Toc258362302)

[10.0 STATISTICAL CONSIDERATIONS 23](#__RefHeading___Toc258362303)

[10.1 Sample Size Justification 23](#__RefHeading___Toc258362304)

[10.2 Statistical Analysis: 25](#__RefHeading___Toc258362305)

[11.0 STUDY LIMITATIONS 28](#__RefHeading___Toc258362306)

[12.0 STUDY TIMELINE 29](#__RefHeading___Toc258362307)

[13.0 ROLES AND RESPONSIBILITIES 29](#__RefHeading___Toc258362308)

[14.0 ADMINISTRATIVE PROCEDURES 30](#__RefHeading___Toc258362309)

[14.1 Study Coordination 30](#__RefHeading___Toc258362310)

[14.2 Study Site Monitoring 30](#__RefHeading___Toc258362311)

[14.3 Protocol Compliance 30](#__RefHeading___Toc258362312)

[14.4 Study Records 30](#__RefHeading___Toc258362313)

[15.0 BUDGET 33](#__RefHeading___Toc258362314)

[APPENDIX I: CONSENT FORM 34](#__RefHeading___Toc258362315)

[APPENDIX II: ELIGIBILITY CHECKLIST 38](#__RefHeading___Toc258362316)

[APPENDIX III: BASELINE QUESTIONNAIRE 39](#__RefHeading___Toc258362317)

[APPENDIX IV: QUESTIONNAIRE AT DAY 42 40](#__RefHeading___Toc258362318)

[APPENDIX V: DRAFT TEXT MESSAGES 41](#__RefHeading___Toc258362319)

# ABSTRACT

Male circumcision (MC) reduces, by more than half, the risk of HIV-1 acquisition. WHO and UNAIDS recommend that “*male circumcision should be recognized as an efficacious intervention for HIV prevention especially in countries and regions with heterosexual HIV epidemics and low male circumcision prevalence.*[1]” As a result, programs have been introduced and scaled up for voluntary medical male circumcision. Kenya leads with the largest expansion of services. Early resumption of sexual intercourse after MC may have deleterious effects, including higher rates of post-operative surgical complications, and higher HIV acquisition among females in couples that resume sexual activity before certified wound healing. In the context of rapid scale-up of MC, adherence to post-operative clinic appointments allows clinicians to assess wound healing and to deliver risk reduction counseling. Abstinence from sexual intercourse before complete wound healing would reduce the rate of post-operative adverse events and minimize the risk of HIV transmission from HIV-infected men to their uninfected female partners. To our knowledge, the effect of reminders delivered via text messaging to promote adherence to clinic visits and abstinence after MC has not been investigated. We propose a randomized controlled trial in which men who will have undergone voluntary medical male circumcision at selected sites in Kisumu will be randomized to receive either the intervention (context-sensitive text messages after circumcision) or the control condition (usual care). This study seeks to determine (a) the effect of regular text messages sent to men after circumcision on attendance of the scheduled 7-day post-operative clinic visit versus usual care; (b) the proportion of men who resume sexual activity before 42 days post-procedure after receiving regular text messages versus usual care within the 42 days post-circumcision; and (c) to identify potential predictors of failure to attend the scheduled 7-day post-operative visit and early resumption of sexual intercourse.

# LIST OF ACRONYMS

| AE | Adverse Experience |
| --- | --- |
| AIDS | Acquired Immunodeficiency Syndrome |
| CI | Confidence Interval |
| CMR | Center for Microbiology Research |
| CONSORT | Consolidated Standards of Reporting Trials |
| ERC | Ethical Review Committee |
| FWA | Federalwide Assurance |
| HIV | Human Immunodeficiency Virus |
| ID | Identification number |
| IRB | Institutional Review Board |
| KEMRI | Kenya Medical Research Institute |
| MC | Male Circumcision |
| MOH | Ministry of Health |
| NRHS | Nyanza Reproductive Health Society |
| NIH | National Institutes of Health |
| OPRR | Office of Protection from Research Risks |
| PI | Principal Investigator |
| SAE | Serious Adverse Experience |
| SMS | Short Messaging Service |
| STD | Sexually Transmitted Disease |
| UNAIDS | Joint United Nations Program on AIDS |
| UNIM | A partnership between the Universities of Nairobi, Illinois, and Manitoba (PI Robert C. Bailey) |
| WHO | World Health Organization |

# INTRODUCTION

## Background

The epicenter of the HIV epidemic remains in sub-Saharan Africa, with 1.9 million new infections in 2008 [2]. Male circumcision (MC) reduces, by more than half, the risk of HIV-1 acquisition [3-5]. WHO and UNAIDS recommend that “*male circumcision should be recognized as an efficacious intervention for HIV prevention especially in countries and regions with heterosexual HIV epidemics and low male circumcision prevalence.* [1]” As a result, programs have been introduced and scaled for voluntary medical male circumcision in several sub-Saharan countries, with Kenya leading with the largest expansion of services (approximately 40,000 MCs done by October, 2009) [6].

Early resumption of sexual intercourse after the MC may have deleterious effects. A study of adverse events related to MC found that post-operative surgical complications occur at higher rates among men who resume sexual intercourse before wound healing is complete than among those who delay resumption of intercourse [7]. In a study assessing the effect of MC on male to female transmission of HIV, higher HIV acquisition occurred among females in couples who resumed sexual activity early (before certified wound healing) compared to those who did not resume sex early [8].

In the context of rapid scale-up of MC, adherence to post-operative clinic appointments is important. It gives clinicians an opportunity to assess wound healing, and provides additional opportunities to deliver risk reduction counseling. Abstinence from sexual intercourse before complete wound healing is vital in order to reduce the rate of post-operative adverse events in both HIV infected and uninfected men, as well as to minimize the risk of HIV transmission from HIV-infected men to their uninfected female partners. To our knowledge, the effect of reminders delivered via SMS to promote adherence to clinic visits and abstinence after MC has not been investigated.

## Benefits of delayed resumption of sex after MC

Delayed resumption of sex after MC potentially reduces the risk of HIV acquisition during the wound healing period. During the healing period, men who engage in sexual activity may be at an increased risk of acquiring HIV infection, though studies on this question remain inconclusive. One study found that sex early after circumcision (less than 42 days after the procedure) was not associated with increased risk of HIV seroconversion, but concluded that the study was underpowered [9]. The authors also concluded that the possibility of increased risk of HIV acquisition still remains. Further, these results were obtained under circumstances that included intensive counseling for trial participants to delay sexual activity until wound healing. Certainly, there is biological plausibility for an increased risk of acquiring infections, including HIV, from sex occurring before wound healing.

For HIV-positive men in discordant relationships, there is the added advantage of reduced risk of transmission to their female partners [8]. A significant reduction in the rates of post-operative surgical complications has also been documented for both HIV-infected and uninfected men who delay resumption of sex [7].

## Counseling for MC

During the conduct of the randomized trials of MC for HIV prevention, participants received intensive counseling on HIV/STI prevention at each trial visit. In the Kisumu trial, for example, risk-reduction counseling was individually-tailored for each participant at every visit [4]. In the setting of programmatic MC in Kenya, counseling is less intensive than was the case in the trials. There is a need to find ways of reinforcing HIV prevention counseling among men who have undergone circumcision. To our knowledge, no interventions to address this gap have been investigated. We therefore propose to compare the current standard of care to text messaging as a simple, affordable and appropriate intervention.

The overall aim of this intervention is to ensure that circumcised men have additional opportunities to receive HIV prevention messages most pertinent to their circumcision status. Ensuring that circumcised men return for the scheduled 7-day follow-up visit should provide additional opportunity for counseling, as well as ensure that clinicians assess wounds for healing. Regular text messages sent after circumcision should also fortify HIV prevention messages already received at the clinic. We hypothesize that this intervention will lead to an increased rate of return for the scheduled 7-day post-operative visit, and a reduced rate of early resumption of sexual intercourse before wound healing.

# RATIONALE

## Studies of medical and behavioral interventions delivered via phone

The use of reminders delivered via communications technology enhances the effectiveness of medical interventions. A randomized controlled trial comparing diabetes control among patients who transmitted their blood glucose levels to a clinic via phone (treatment group) to those who physically went to the clinic with results (control) found better blood glycemic control in the treatment group (lower HbA1c) [10]. In the United States, an RCT compared follow-up phone call by a pharmacist 2 days after discharge from hospital to no phone call and found the phone call group more satisfied with discharge instructions (86% vs. 61%, *P* = 0.007) [11]. Another RCT found a significant improvement in adherence to antihypertensive medication among patients who used a telephone reminder system in addition to usual medical care (P = .03), with a significantly lower mean diastolic blood pressure (P = .02) [12].

## Telephone reminders and adherence to clinic appointments

With regard to improving adherence to scheduled clinic appointments, telephone reminders lead to higher attendance rates. A study among attendants at an adolescent clinic found that the attendance rate was significantly increased when a telephone reminder was given one day before a clinic appointment, compared to no reminder [13]. SMS text reminders have also been shown to significantly improve clinic attendance. A cohort study in Australia found the rate of failure to attend clinics much lower among patients who received an SMS reminder (14.2% v 23.4%; *P* < 0.001) [14].

## Mobile telephone technology in developing countries

Most studies on the topic of delivery of medical and behavioral interventions using telephone systems have been conducted in developed countries that have a high coverage of telephones with robust communication networks. However, the recent rapid increase in mobile telephony in developing countries presents a new avenue for similar interventions. In Kenya, mobile cellular subscription has grown from 0.4 per 100 people in 2000 to 30.2 per 100 people in 2007, with 77% of the population covered by a mobile cellular network [15]. A 2006 survey in an economically-disadvantaged part of Nairobi found that 89% of respondents had access to a mobile phone [16]. Respondents in that survey also found mobile phones acceptable for receiving HIV-related information. In South Africa, mobile telephone technology used to monitor adherence to highly active antiretroviral therapy was found to be acceptable to both patients and providers [17].

The feasibility of using mobile phones in public health interventions in developing countries has been demonstrated. A study in Peru demonstrated the feasibility of using mobile telephony to collect data on adverse effects caused by metronidazole therapy for treatment of sexually transmitted infections in remote communities [18]. SMS text messaging has also been employed with much success in improving adherence to treatment in a South African tuberculosis program [19].

The Nyanza province of Kenya has a high HIV prevalence (14.9%) and low prevalence of male circumcision (48.2%) [20]. In Kisumu, the largest town in Nyanza, only about 10% of adult Luo males are circumcised [4]. This has made it the province of priority in terms of implementing voluntary medical male circumcision programs. Since the launch of the Kenya Ministry of Health’s Voluntary Medical Male Circumcision program, there has been a high uptake of MC services in this region. This rapid growth in the MC program is likely, in the long term, to result in a significant reduction in HIV incidence and prevalence with a public health impact. In order to insure these gains, it would be important to investigate strategies to mitigate any potential hindrance.

## Study Generalizability

This study will help to evaluate a strategy to improve adherence to post-operative clinic visits and reduce early resumption of sex after MC. If proven superior to usual care, the proposed intervention (regular, context-based text-messaging) can be easily integrated into existing healthcare systems in resource-limited settings. Findings from this study will provide randomized trial evidence that will inform HIV prevention program planners and implementers in Kenya and beyond.

# STUDY OBJECTIVES AND HYPOTHESES

The general objective of this study is to evaluate an intervention that will promote adherence to post-operative clinic visits, and open up additional opportunities for circumcised men to receive HIV prevention messages (at the clinic and on their mobile phones), such as the benefits of abstinence until wound healing, and the harmful effects of early resumption of sexual intercourse.

## Specific Objectives

**Objective 1:** To determine the effect of regular, context-sensitive text messages sent to men after undergoing circumcision versus usual care on attendance of the scheduled 7-day post-operative clinic visit, in a randomized controlled trial.

**Hypothesis 1:** We hypothesize that men who receive the SMS intervention will have higher rates of attendance at the 7-day post-operative visit, compared to those receiving usual care.

**Objective 2:** To determine the proportion of men who resume sexual activity before 42 days post-procedure (wound healing is expected to be complete in the majority of circumcised men within 42 days [7]) after receiving regular, context-sensitive text messages versus usual care within the 42 days post-circumcision.

**Hypothesis 2a:** We hypothesize that men who receive the SMS intervention will report higher rates of abstinence from sex during the 42 days post-circumcision, compared to those receiving usual care.

**Hypothesis 2b:** We also hypothesize that, among those who resume sex before 42 days post-procedure, those who receive the messages resume sex later than those who do not.

## Secondary Objective

To identify potential predictors of failure to attend the scheduled 7-day post-operative visit, and also to identify potential predictors of early resumption of sex after MC.

## Study Outcome Measures

### Primary Outcome Measures

The primary outcome measure for objective 1 is the proportion of men failing to return for a post-operative clinic visit at 7 days. This proportion will be determined by examining each participant’s clinic records after their 7^th^ post-operative day. Adherence to this clinic visit will be analyzed as a dichotomous variable. Participants will be considered to have ‘attended’ or ‘not attended’ the clinic visit after the elapse of 3 days after the scheduled 7-day visit.

The primary outcome measure for objective 2 is the proportion of men who report resumption of sexual activity before 42 days post-circumcision. This proportion will be determined by self-report using a brief questionnaire delivered via SMS. The analysis will be as a dichotomous variable, with participants considered as having either ‘resumed’ or ‘not resumed.’ Among men who resume sex before 42 days post-circumcision, we will also compare the time to resumption by study arm.

### Secondary Outcome Measures

Reasons for failure to attend the scheduled 7-day post-operative visit will be evaluated as a secondary outcome measure. These reasons will be determined by administering a questionnaire in person at baseline to all participants, followed by a similar questionnaire by phone call at 42 days to a sub-sample of participants who fail to return for the 7-day visit.

# STUDY DESIGN

## Overview of Study Design

The proposed study is a randomized controlled trial. Men who will have undergone voluntary medical male circumcision will be approached for study enrollment. Participants will be randomized to receive either the intervention (context-sensitive text messages after circumcision) or the control condition (usual care). Participants in the intervention arm will receive text messages as described in section 5 below. All participants will be followed up for a total of 42 days.

## Study Setting

This study will be conducted in Kisumu. Kisumu is the third largest city in Kenya and is located in Nyanza province, which is in western Kenya on Lake Victoria. Kisumu city has a population of approximately 400,000. The majority of Kisumu residents belong to the Luo ethnic group, one of the largest tribes in Kenya. The Luo are traditionally a non-circumcising community. The fishing community in Kisumu has a very high prevalence of STIs including HIV [21, 22]. Nyanza is also the province in Kenya where programmatic implementation of voluntary medical male circumcision has been launched by the Ministry of Health and is being brought to scale.

## Study Population

Participants for this study will be recruited from among men who will have undergone circumcision at selected sites in Kisumu East and West operated by the Nyanza Reproductive Health Society. The precise clinics where we will conduct the study will be determined once we are in the field, but the performance site for the study is the Nyanza Reproductive Health Society, which performs male circumcision at 16 fixed and mobile sites in Kisumu East and West.

## Inclusion Criteria

Participants who meet the following criteria will be eligible for enrollment:

1. Male
2. 18 years or older
3. Have undergone circumcision on the day they are screened for the study
4. Currently own a mobile phone with text-messaging capability, and
5. Have the mobile phone in their possession at the time of enrollment
6. Able and willing to respond to a questionnaire administered via a phone call

## Exclusion criteria

Participants who meet the following exclusion criteria will **not** be eligible for enrollment:

1. Prior participation in a study on male circumcision
2. Currently participating in other ongoing research studies
3. Any medical condition or situation exists such that study participation would not be in the man’s best interest, as determined by the investigator

# METHODOLOGY

## Treatment Assignment Procedures

### Randomization Strategy

All enrolled participants will be randomized to receive either the intervention condition (text message) or control condition (no text message; standard of care) in a ratio of 1:1 after giving informed consent for the study.

### Sequence generation

A block randomization scheme with variable block sizes will be generated using STATA 10.1 ralloc.ado module v3.5.2 [23]. This will ensure approximately equal sample sizes, and that participants and study staff cannot anticipate assignment to either group. Treatments will be allocated in 1:1 ratio. All the study investigators and staff will be blinded to the block number, block size and sequence in the block. The treatments will be assigned via pre-prepared sealed, opaque envelopes and the envelopes will be ordered in the sequence of treatment assignments generated by the STATA code. Once eligibility for randomization has been determined, the first available allocation envelope will be assigned to the study subject. The subject will be randomized to the treatment arm indicated inside the envelope.

### Allocation concealment

As potential subjects are identified, a research assistant will be notified to assess eligibility. Once a subject is deemed eligible for enrolment, has given the necessary informed consent, has been enrolled, has responded to a brief questionnaire, and has had his demographic information abstracted from his clinic records, the research assistant will issue the next in a series of sequentially numbered, sealed, opaque envelopes containing group assignment [24]. The participant’s clinic number will be written on the envelope, and the participant will be required to open and read the treatment assignment in the presence of the research assistant, who will then enter the group assignment into a database and store the envelope. If a participant is unable to read, the research assistant will read the group assignment on his behalf.

### Implementation of Randomization Procedures

A biostatistician assigned to the study from the Biometrics Core of the Center for AIDS Research at the University of Washington will carry out randomization procedures in Seattle, USA. The biostatistician will not be involved in any other aspects of conducting the study. The sequentially numbered, opaque sealed envelopes containing group assignment will be shipped to by the biostatistician from Seattle to the study coordinator in Kisumu via FedEx. The study biostatistician will retain the key to treatment assignments. The study coordinator will be on site every day to ensure compliance with the study protocol.

### Blinding

Because of the nature of this intervention, it will not be possible to blind the participants to the group assignments. Clinicians and nurses conducting MC procedures and follow-up will, however, not be aware of study group assignment.

## STUDY INTERVENTION/TEXT MESSAGING PROTOCOL

### Schematic of text messaging protocol:

**Demographic Information Module**

Abstract demographic information from participant’s clinic file into electronic database

**Study Registration Module**

SMS-based registration: SMS containing *Study Arm, time to send SMS, preferred language*

**Text Messaging Module (days 8, 14, 21, 28, 35, 41 and 42)**

**Response within 1 week**

No Response

Loss to follow-up

**Enrollment Module (SMS-based registration)**

Assign Study ID number

Complete electronic enrollment form

Participant found eligible; informed consent obtained

**Day 7 (+/- 3 day window)**

Abstract data from participant’s clinic records into electronic database

**Day 42 (+ 7 day window)**

Administer questionnaire via phone call

Randomization to study arms

**Allocation to intervention (n=600)**

**Allocation to control (n=600)**

**Text Messaging Module (days 1,2,3,4,5,6,7)**

### Rationale for Selecting Intervention and Control Conditions

Several potential study arms were considered before finally selecting the intervention (personal phone and text messaging) and control (personal phone and no text messaging) groups. The table below summarizes the pros and cons of several different intervention and control choices:

|  | **PROS** | **CONS** |
| --- | --- | --- |
| **Intervention:**  Personal Phone + SMS  **Control:**  Personal Phone + No SMS (usual care) | - Removes study-issued phone as a potential reminder (isolates effect of SMS alone) - Minimal extra cost for scale up - Easier to integrate to existing programs and bring to scale - Less expensive study - Closer to an effectiveness study as relates to men with phones | - Excludes men without phones (generalizable only to men with phones) - May substantially reduce size of eligible population due to phone ownership - Exposing sensitive personal information if phone is shared and not owned |
| **Intervention:**  Study Phone + SMS  **Control:**  Study Phone + No SMS | - Phone acts as incentive for study retention for both groups - Isolates the effect of SMS | - Study phone a powerful reminder - Affordable study but adds substantial cost to MC - Limits ability to disseminate - Unlikely to be implemented widely due to cost (everyone may say that they don’t have a phone) |
| **Intervention:**  Study Phone + SMS  **Control:**  No Phone + No SMS + Study Phone at 42 days | - Study phone acts as incentive for study retention for intervention arm | - Study phone itself is a powerful reminder - Affordable study but adds substantial cost to MC - Hard to tell if any benefits are due to SMS or phone |
| **Intervention:**  Study Phone + SMS  **Control 1:**  Study Phone + No SMS  **Control 2:**  Usual care (No Study Phone + No SMS) | - Gives study ability to analyze effect of phone vs. effect of SMS - If successful, would guide programs on whether to buy phones or not for best results | - Larger sample size - Affordable study but adds substantial cost to MC |
| **Intervention:**  Study Phone + SMS  **Control 1:**  Study Phone + Sham SMS  **Control 2:**  Study Phone + No SMS  **Control 3:**  Usual care (no phone + no SMS) | - Ability to detect whether differences are due to **content** of SMS | - Larger sample size - Study phone a powerful reminder - Sham SMS also a strong reminder - Affordable study but adds substantial cost to MC |

### Product Acquisition/Software Development

A robotic text messaging software will be developed in conjunction with a contracted third party, Dimagi, Inc. (Charlestown, MA). The software will be built on Dimagi’s existing RapidSMS platform, an open source framework for SMS and web integrated applications [25].

We will work with Dimagi to build into RapidSMS functionality that will allow the software to send out pre-programmed text messages to the intervention group at the time of day and in the language that participants will have determined as most convenient for receiving text messages. Dimagi, Inc. will be responsible for installing the software on site in Kenya and for testing.

Schematic depicting incoming and outgoing flow of SMS

|  |
| --- |
|  |

### Text Message Formulation/Scripting

Text messages will follow a pre-defined script (appendix V) and will be limited to a template of not more than 160 characters (the maximum number of characters allowed for a single text message). The contents of each message as provided in appendix V are draft messages. We may amend the actual wording (before study initiation) based on responses after field testing as well as expert advice from our behavioral scientists.

Text messages will be sent in the participant’s preferred language from a choice of English, Swahili or Dholuo. The text messages will not include the participant’s name, clinic number or HIV status in order to maximize confidentiality.

### Text Messaging Regimen

The text messaging software will be programmed to send out one text message daily to participants in the intervention arm for the first seven days post-procedure. After the first seven days, a text message will be sent on each of days 8, 14, 21, 28, 35, 41 and 42. Each message will be different depending on which post-procedure day it will be for the recipient (per appendix V). Individual text messages will be sent out at the time of day, and in the language, that a participant will have determined as most convenient for receiving text messages.

Participants from either arm who have questions or concerns about their circumcision will call the clinic hotline number that is routinely issued to all men who undergo circumcision. The text messaging software will be set up to handle only text messages and not voice calls.

### Shared Phones

Cases may arise where participants in the intervention arm share their phone with other people. The study will NOT send out personal health information such as HIV status as part of any text message. All study procedures will be thoroughly explained to participants. Participants will be given examples of text messages they may receive and will be asked to give consent only if they find the messages acceptable to be sent to their phone, whether shared or not.

### Cost of Intervention

Because the SMS system is only one way, there is no cost to the study subjects except the cost of sending the registration message. At enrollment, we will load each participant’s phone with airtime worth twenty Kenya shillings (KSh.20) immediately prior to registration. This system will ensure that participants do not bear any costs related to sending or receiving text messages related to this study.

### Storage and Security of Software/Hardware for Delivering Intervention

The computer hardware and software used to house the robotic text messaging software will be kept in a secure room with access limited only to authorized staff. All computers and databases will be password-protected with limited access. Daily backup of the data will be done by a designated study staff.

# STUDY CLINIC VISITS AND PROCEDURES

Participation in this study requires only a single clinic visit at 7 days, followed by a brief questionnaire at 42 days delivered via a phone call.

## Screening Visit

As part of the Kenya Ministry of Health’s MC program, community mobilization is done using a variety of methods, including radio announcements, posters, fliers, mobile vans and drama shows. Men interested in undergoing MC then visit clinics of their choice for education on male circumcision, HIV testing and counseling, booking according to the clinic schedule, and consent and performance of the procedure. Mobilization for this study will be done from among men who will have undergone circumcision at selected sites in Kisumu East and West operated by the Nyanza Reproductive Health Society. All men who meet the study inclusion criteria will be invited to participate.

After the circumcision procedure, patients usually have a 30-minute recovery period. During this time, patients are monitored for effects of surgical trauma and any early complications that may arise. At the end of this recovery period, post-operative care instructions are given verbally and in writing, and the patient discharged.

For this study, study staff will approach men for possible enrollment immediately after discharge but just before a potential participant leaves the recovery room. Study staff will introduce the study to a potential participant by describing the purpose of the study, the anonymity of study data and the participant’s right not to answer any questions. Men who agree to be screened will be led to a separate private counseling room away from the recovery room in order to determine eligibility using the eligibility checklist (appendix 2).

## Enrollment Visit

Enrollment into the study will occur on the same day as screening. While still in the private counseling room, participants who are found to be eligible will be offered enrollment into the study. Study procedures will then be described in detail and participants asked to provide written informed consent prior to enrollment. The informed consent form will also request participants’ permission to access their MC clinic records. The informed consent process is described in section 10.1 below.

After the informed consent process, study staff will abstract demographic information from a participant’s clinic file. Data collected will include *client number, mobile phone number, date of MC, date of birth, ethnic origin, district of residence, sub-location/estate of residence, education level, employment status, marital status, religion, age at first sex, number of sex partners in last month, whether subject has ever been tested for HIV, whether client was given condoms, how client learnt about MC services, referral source, and cadre of person who performed procedure.* HIV status and/or test results will NOT be collected as part of this study. Study staff will also administer a brief questionnaire to all participants (appendix III)*.*

Following enrollment, participants will be randomized to either of the study arms. Randomization procedures are described in detail in section 3.5 above. Participants’ mobile phone numbers will be confirmed using an SMS-based registration system. This will involve sending a text message from a participant’s phone to a phone number controlled by our robotic software. The text message will contain the time of day that they find most suitable for messages to be sent to them as well as the language in which they prefer to receive text messages (from a choice of English, Swahili, or Dholuo). Upon receiving this initial text message, the software will decipher the contents and also store the subject’s phone number. The software will have been pre-programmed to send specific text messages to those in the intervention arm, one on each day at the time and in the language specified by the subject for the first 7 days post-procedure. Details are provided in section 5 below. Participants will be reminded of all study procedures again before leaving the clinic.

## Follow-up Visit

According to the Kenya national guidelines for comprehensive male circumcision services [26], circumcised men are required to visit the clinic at 7 days post-procedure. At this visit, the wound is examined for healing progress, signs of infection, and signs of other post-operative surgical complications. Additional examinations and/or referral are done based on the man’s symptoms. All clinic procedures are documented in the patient’s clinic records. These clinical procedures are not study procedures. Our study will only be interested in extracting data from clinic records.

After the 7^th^ post-operative day, study staff will check participants’ clinic records to ascertain their attendance of this visit and to allow for assessment of study outcome measures. A 3-day window before and after the date of the scheduled visit is allowed. Study staff will abstract data from participants’ clinic records. The data collected will include: *date of visit, date of circumcision, type of visit (routine/client-initiated/physician-scheduled), adverse events (event, grade, treatment), resumption of sexual intercourse, days since resumption, and satisfaction with MC procedure*. HIV status and/or test results will NOT be collected as part of this study.

After the scheduled 7-day post-operative visit, participants in the intervention arm will receive one text message on each of the following days post-procedure: days 8, 14, 21, 28, 35, 41 and 42.

## Final Study Visit (Virtual Visit)

At 42 days post-procedure, a final intervention text message will be sent to participants randomized to the intervention arm. After the final SMS at day 42, participants in both arms will be interviewed via phone call to determine outcome data for our second primary objective (appendix IV). All participants will also be invited to visit the clinic should they wish to receive free condoms

At conclusion of the randomized controlled trial, a subset of participants who failed to return for the scheduled 7-day post-operative visit will be invited to take part in a phone interview to ascertain reasons for failure to return. A brief questionnaire (similar to the baseline questionnaire, appendix III) will be administered via phone interview. Items in this questionnaire will allow for assessment of secondary study outcome measures. Responding to this questionnaire will terminate study participation.

## Study Withdrawal for Participants

Participants will be free to withdraw from the study at any time by presenting to the study clinic and indicating their desire to withdraw. Reasons for study withdrawal will be ascertained. Where possible, information on study primary and secondary endpoints will be evaluated at the time of study withdrawal. No further messages will be sent, or phone calls made, to a participant who deliberately withdraws from the study.

# STUDY ADVERSE EVENTS

Due to the nature of the intervention, no adverse events are expected that are related to this study. Participants who experience any adverse events related to their circumcision will be expected to seek treatment as per the Kenya national guidelines of male circumcision.

Participants will be reminded not to use mobile phones while driving or riding.

# ETHICAL CONSIDERATIONS

## Informed Consent

All eligible men will be asked to sign and date an informed consent form prior to enrolment. The consent forms shall describe in detail the study procedure in the language the subject is comfortable with (English, Swahili or Dholuo). The subject will be asked to read and review the document. If a subject cannot read, the consent form will be read to them. The study will then be explained to the subject by a study staff, who will also answer any questions that arise. A copy of the informed consent document will be given to the subjects for their records.

## Ethical Approval

All study participants will be fully informed of the study procedures as described above. Ethical approval for this study will be sought from the Kenya Medical Research Institute’s (KEMRI) Ethical Review Committee, the University of Washington’s Human Subjects Division, and the Institutional Review Board at the University of Illinois at Chicago. KEMRI’s review system will involve an initial review of the protocol by the Center for Microbiology Research Scientific Committee. Upon approval, the protocol will be submitted to the KEMRI Scientific Steering Committee to be reviewed for scientific merit. If approved, the protocol will be forwarded to the KEMRI/National Ethics Review Committee for final ethical approval. At the University of Washington, the study proposal will undergo peer review by the International AIDS Research and Training Program before being forwarded to the Human Subjects Division for ethical review.

## Protecting Privacy and Confidentiality

All education, counseling and testing will be done as stipulated in the Kenya national guidelines for safe voluntary medical male circumcision. All participants will be identified using an identification code that will be the same as the client number assigned to them at the clinic. None of the clinic procedures are study procedures. Our study specifically seeks to obtain approval to access client records and to link the data to our study identification number.

All data, including the computer containing the automated SMS software and databases, will be locked in a secure room with access restricted only to authorized study personnel. Similarly, all computers containing study data will be password protected and only accessible to authorized study staff.

## Potential Risks of Proposed Research to Study Subjects

We anticipate minimal risk to study participants in this study. We do not intend to collect any data about participants’ HIV status. No biological specimen will be collected.

There exists the potential for exposing a participant’s personal information to others, especially if phones are shared. However, the study will not send out any sensitive personal health information. Further, study procedures will be thoroughly explained to participants and they will be consented only if they fully understand study procedures and find the messages acceptable to them. Participants are also allowed to exit from the study at any time.

Patient anonymity may be compromised since the phone numbers to which messages are sent are essentially personal identifiers. However, the computer on which these phone numbers will be stored will be accessible only to specifically designated study staff. Every effort will be made to safeguard subjects from future contact. The electronic databases will not contain participant names. Health information will be protected by coding all information required for the study and keeping the coded data separate from clinic records. The list of coded and uncoded information (linkage sheet) will also be kept separately. This linkage sheet will be maintained for a maximum of five years, after which all links between coded and uncoded data will be broken. All databases will be password protected and accessible only to authorized study staff.

Because we will be accessing clinic records, there is a risk of breach of confidentiality. All study staff will have undergone training in order to ensure that confidentiality of patient data is maximized.

## Potential Benefits of Proposed Research to Study Subjects and Others

During the administration of the final questionnaire, participants will be invited to visit the clinic should they wish to receive free condoms.

Knowledge gained from this study will help inform policy makers and HIV prevention programs on devising strategies that can improve adherence to the 7-day post-op visit, reduce early resumption of intercourse after MC, and reduce the frequency of unprotected sexual intercourse.

This study will also provide critical information on the feasibility of using mobile telephony-based technology for public health interventions

# DATA COLLECTION AND MANAGEMENT

## Data Collection

Enrollment data will be directly entered into an electronic database as it is collected during the enrollment visit. A study data clerk will be assigned to enter all such data.

All data collected at the 7-day visit will be abstracted from clinic records directly into an electronic database by a study data clerk. Data abstraction for the 7-day visit will be a continuous process and will occur immediately after the expiry of the 7-day post-MC period. We will develop electronic forms that will allow for this process.

For the final follow-up virtual visit (interview at day 42), responses from the questionnaire administered via phone call will be recorded by study staff into an electronic database. If a participant fails to respond to the phone call, study staff will retry twice daily for one week (7 days). If a response is not received by this time, the participant will be considered lost to follow up due to inability to collect data on the final outcome measure. Data entry will be done by designated study staff that will have undergone training.

## Data Storage and Security

All study computers and records will be stored in a secure room with access limited only to authorized staff. All computers and databases will be password-protected with limited access. Daily backup of the data will be done by a designated study staff onto an external hard drive and onto a server at the University of Washington. The external hard drive will be stored in a separate building that houses the data management center for the Research Care and Training Program in Kisumu (Director, Dr. Elizabeth Bukusi).

# STATISTICAL CONSIDERATIONS

## Sample Size Justification

We calculated the sample sizes for each specific aim using the methods described by Dupont WD et al. [27] using the following formula:


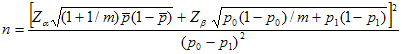


Where
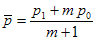


n = desired sample size
Z_α_ = standard normal deviate (z-score) corresponding to the 5% significance level (Z_α_ = 1.96)
Z_β_ = standard normal deviate (z-score) corresponding to the acceptable type II error rate (Z_β_ = 1.28 for Objective 1; Z_β_ =0.84 for Objective 2)
p_0_ = probability of event in controls
p_1_ = probability of event among participants in the intervention arm, assuming the intervention works as hypothesized
m = number of control subjects per experimental subject

**Objective 1:** To determine the effect of regular, context-sensitive text messages sent to men after undergoing circumcision versus usual care on attendance of the scheduled 7-day post-operative clinic visit. We hypothesize that men who receive the SMS intervention will have higher rates of attendance compared to those receiving usual care.

Assuming that the proportion of men failing to return for the 7-day post-operative visit is 0.57 among the control group (Prof. Robert Bailey, Nyanza Reproductive Health Society: personal communication), and a 2-sided test with a significance level of 0.05, with **600 men in each arm** we will have at least **90% power** to detect a relative risk between the two arms of **0.83** or larger.

**The following table summarizes the range of sample sizes required with different assumptions of the true relative risk of failure to return for subjects in the intervention arm relative to controls:**

|  | RR = 0.5 | RR = 0.6 | RR = 0.7 | RR = 0.8 | **RR = 0.83** | RR = 0.9 |
| --- | --- | --- | --- | --- | --- | --- |
| Sample Size (per arm) **90% power** | 61 | 98 | 177 | 402 | **557** | 1604 |
| Sample Size (per arm) **80% power** | 46 | 74 | 133 | 301 | 416 | 1199 |

**Objective 2:** To determine the proportion of men who resume sexual activity before 42 days post-procedure after receiving regular, context-sensitive text messages versus usual care within the 42 days post-circumcision. We hypothesize that men who receive the SMS intervention will report higher rates of abstinence from sex during the 42 days post-circumcision.

The proportion of circumcised men who reported early resumption of sexual activity in the South African MC trial was **0.225** [9]. This trial was selected because the counseling regimen used was designed to approximate programmatic implementation of MC. Assuming that the proportion of men resuming early sexual intercourse is 0.225 among the control group, a 2-sided test with a significance level of 0.05, beginning with 600 men in each arm and about 11% loss to follow-up at 42 days, with **532 men** remaining in each arm we will have at least **80% power** to detect a relative risk between the two arms of **0.70** or larger.

The following table summarizes the range of sample sizes required with different assumptions of the true relative risk of failure to return for subjects in the intervention arm relative to controls:

|  | RR = 0.50 | RR = 0.60 | RR = 0.65 | **RR = 0.70** | RR = 0.75 | RR = 0.80 |
| --- | --- | --- | --- | --- | --- | --- |
| Sample Size (per arm) **90% power** | 231 | 381 | 510 | 712 | 1048 | 1674 |
| Sample Size (per arm) **80% power** | 173 | 285 | 382 | **532** | 783 | 1251 |


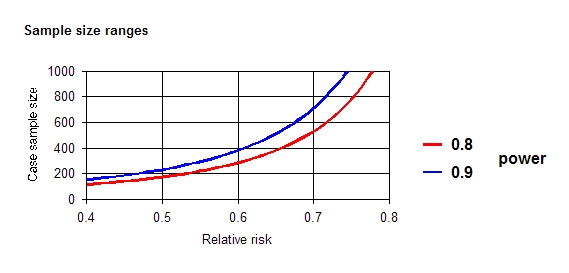


Our enrollment target will therefore be 1,200 participants (600 per arm).

## Statistical Analysis:

### Descriptive analyses

**Trial Profile**

The flow of participants through the study will be reported using a “CONSORT” diagram [28]. We will report the number of men screened, those who met inclusion criteria, those that were excluded and reasons for exclusion, and the number randomized by study arm. All participants for whom the primary outcomes will not be available will be reported as lost to follow-up.

**Baseline data:**

A table will be used to summarize the comparison of baseline characteristics for men in both study arms. This table will include descriptive statistics of the quantitative variables (mean, median, variance, range, IQR, standard deviations etc) as well as frequencies for the categorical variables. This table will serve to describe the study population, stratified by study arm, and also to verify that potential confounders were well balanced by randomization. Baseline characteristics that are not balanced between the study arms will be adjusted for in subsequent sub-group analyses. Baseline analyses will also analyze the extent and distribution of missing data.

Baseline characteristics that are continuous will be compared using Wilcoxon and t-tests. Characteristics that are categorical will be compared using Chi-square test. Analyses will be done using Stata (StataCorp. 2007. *Stata Statistical Software: Release 10*. College Station, TX: StataCorp LP.).

### Inferential analyses

The primary analysis will follow the intent-to-treat principle, that is, we will analyze the arm to which the subject was randomly assigned. All tests will be conducted at the 5% significance level (two-sided).

**Testable Hypothesis 1:**

A Chi-square test will be used to compare the proportions of men attending the scheduled 7-day post-operative visit by study arm. A follow-up analysis comparing the proportions attending 7-day visits will also be done that further adjusts for variables that were imbalanced between arms at baseline.

**Testable Hypothesis 2:**

A chi-square test will be used to compare the proportions of men resuming early sexual intercourse (defined as resuming sex before 42 days post-procedure) by study arm. A follow-up analysis comparing the proportions resuming early sexual intercourse will also be done that further adjusts for variables that were imbalanced between arms at baseline.

Among men who report early resumption of sexual intercourse, the time to resumption (in weeks from MC procedure to resumption) of sexual intercourse will be compared by study arm using Kaplan-Meier plots, log-rank tests, and Cox regression. A follow-up analysis comparing the time to resumption will also be done that further adjusts for variables that were imbalanced between arms at baseline.

We will also conduct a multivariable analysis to estimate the effect of resumption of sex as a function of several factors.

Among men who report early resumption of sexual intercourse, the number of sex partners (binary; 1 versus 2 or more) will be compared by study arm using a Chi-Square test. A relative risk and 95% confidence interval will be calculated.

Among men who report early resumption of sexual intercourse, the proportion of men reporting unprotected sexual intercourse will be compared by study arm using a Chi-Square test. A relative risk and 95% confidence interval will be calculated.

### Sensitivity Analyses

Men who are not available for the 42-day assessment (lost to follow-up) will not be available for the analyses in objective 2. Baseline characteristics will be compared among those who are and are not lost to follow-up. Another comparison of baseline characteristics will be performed to assess imbalance between randomized groups among only those subjects who are not lost to follow-up; the analyses in objective 2 will be repeated analyzing for the imbalanced variables. Sensitivity analyses will also be performed that “impute” the missing data under various assumptions (e.g. missing as failure, multiple imputation).

### Estimated value "dummy" calculations using predicted data values

| |  | **Fail to attend** | **Attend** | **Total** | | --- | --- | --- | --- | | SMS Group | 100 | 500 | 600 | | Control Group | 300 | 300 | 600 | | Total | 400 | 800 | 1200 | | RR = (100/600)  (300/600)  RR = 0.33  Conclusion: SMS reduces the risk of failure to attend clinic visits by 67% |
| --- | --- | --- | --- | --- | --- | --- | --- | --- | --- | --- | --- | --- | --- | --- | --- | --- | --- |

| |  | **Resumption** | **No Resumption** | **Total** | | --- | --- | --- | --- | | SMS Group | 95 | 505 | 600 | | Control Group | 135 | 465 | 600 | | Total | 400 | 800 | 1200 | | RR = (95/600)  (135/600)  RR = 0.70  Conclusion: SMS reduces the risk of early resumption of sex by 30% |
| --- | --- | --- | --- | --- | --- | --- | --- | --- | --- | --- | --- | --- | --- | --- | --- | --- | --- |

# STUDY LIMITATIONS

Due to the nature of the intervention, it will not be possible to blind participants to study group assignment. However, clinicians and nurses involved in the routine care of the participants will be blinded, unless participants disclose to them.

Resumption of sexual intercourse at 42 days will be recorded based on self-reports. Since there is no feasible biological surrogate marker for this measure, our results may be affected by social desirability bias since participants may have a tendency not to report early resumption of sex.

This study will exclude men who do not own or have access to a mobile phone. There may be some selection bias because younger men as well as poor men are less likely to have phones. This may affect the generalizability of results. However, the majority of men in the age-group targeted by the MC intervention already own mobile phones, or have access to one. Furthermore, mobile phone access in general is rapidly expanding, and we expect this not to be a major limitation.

Contamination: It is possible that men in the intervention arm may share messages with friends receiving usual care. However, it is unlikely that such men will be circumcised on the same day.

If participants share phones with others, there exists the potential for exposing personal information to others. However, the study will not send out any sensitive personal health information. Further, study procedures will be thoroughly explained to participants and they will be consented only if they fully understand study procedures and find the messages acceptable to them.

At day 42, recall bias related to resumption of sexual intercourse may also interfere with accuracy of results. However, because the duration of interest is relatively brief, we expect recall to be accurate.

Lost phones or changed phone numbers: Participants will be asked for an alternate phone number, if available. This number would then be used for the final interview should calls to the primary number fail.

Some participants may seek follow-up care at non-participating centers closer to their homes. Participants will be educated to return to the circumcising centers for all follow-up care. Since the clinic number on a patient’s record is a unique identifier across all MC sites operated by the NRHS, patients who seek care at any one of these sites will be identified and their records captured.

# STUDY TIMELINE

The total period planned for the project is a total of 19 months as shown in the table below:

|  | **2010** | | | | | | | | | | | | **2011** | | | | | | |
| --- | --- | --- | --- | --- | --- | --- | --- | --- | --- | --- | --- | --- | --- | --- | --- | --- | --- | --- | --- |
|  | **Jan** | **Feb** | **Mar** | **Apr** | **May** | **Jun** | **Jul** | **Aug** | **Sep** | **Oct** | **Nov** | **Dec** | **Jan** | **Feb** | **Mar** | **Apr** | **May** | **Jun** | **Jul** |
| Protocol Development |  |  |  |  |  |  |  |  |  |  |  |  |  |  |  |  |  |  |  |
| Software Development |  |  |  |  |  |  |  |  |  |  |  |  |  |  |  |  |  |  |  |
| Study Instruments |  |  |  |  |  |  |  |  |  |  |  |  |  |  |  |  |  |  |  |
| Human Subjects Review |  |  |  |  |  |  |  |  |  |  |  |  |  |  |  |  |  |  |  |
| Study Enrollment |  |  |  |  |  |  |  |  |  |  |  |  |  |  |  |  |  |  |  |
| Study Procedures |  |  |  |  |  |  |  |  |  |  |  |  |  |  |  |  |  |  |  |
| Data Cleaning; Analysis |  |  |  |  |  |  |  |  |  |  |  |  |  |  |  |  |  |  |  |
| Thesis Writing |  |  |  |  |  |  |  |  |  |  |  |  |  |  |  |  |  |  |  |
| Dissemination |  |  |  |  |  |  |  |  |  |  |  |  |  |  |  |  |  |  |  |

# ROLES AND RESPONSIBILITIES

The study is being carried out by Dr. Thomas Odeny as a partial fulfillment of the requirements for the degree of Masters of Public Health (Epidemiology and Global Health) from the University of Washington. Dr. Odeny will have overall responsibility for the operation and management of the study, under the mentorship of Drs Elizabeth Bukusi, Scott McClelland, King Holmes and Robert Bailey.

| **Title** | **Role** | **Responsibilities** |
| --- | --- | --- |
| Study Coordinator | The Study Coordinator is responsible for developing, in conjunction with the co-investigators, the study manual of operations and other supporting documents. The Study Coordinator ensures that the project is delivered on time, within budget, and to the required quality standards. | - Manage and lead the study team. - Manage the coordination of the partners. - Develop and maintain a detailed project plan. |
| Co-Investigators | Mentorship | - Review and approve protocol - Provide guidance on study implementation - Review study procedures from time to time to ensure compliance with protocol |

# ADMINISTRATIVE PROCEDURES

## Study Coordination

The study coordinator will convene monthly conference calls with the study co-investigators/mentors. The agenda for these meetings will include updates on study progress and ensuring compliance with the protocol.

## Study Site Monitoring

The principal investigator (PI) will be at each site at least once a week during the enrollment period, and thereafter at least once a month. The senior co-investigators/mentors may visit the study sites at their convenience. All records will be made available to the appropriate individuals at these visits.

## Protocol Compliance

The study will be conducted in full compliance with the protocol. Any protocol amendments requiring IRB approval will be submitted to the institutional review boards of the University of Washington, KEMRI and the University of Illinois at Chicago.

## Study Records

All study records will be maintained for a period of three years after the completion of the study.
